# An Induced Pluripotent Stem Cell Patient Specific Model of Complement Factor H (Y402H) Polymorphism Displays Characteristic Features of Age‐Related Macular Degeneration and Indicates a Beneficial Role for UV Light Exposure

**DOI:** 10.1002/stem.2708

**Published:** 2017-10-09

**Authors:** Dean Hallam, Joseph Collin, Sanja Bojic, Valeria Chichagova, Adriana Buskin, Yaobo Xu, Lucia Lafage, Elsje. G. Otten, George Anyfantis, Carla Mellough, Stefan Przyborski, Sameer Alharthi, Viktor Korolchuk, Andrew Lotery, Gabriele Saretzki, Martin McKibbin, Lyle Armstrong, David Steel, David Kavanagh, Majlinda Lako

**Affiliations:** ^1^ Institute of Genetic Medicine, International Centre for Life United Kingdom; ^2^ Campus for Ageing and Vitality Institute for Cell and Molecular Biosciences and Institute for Ageing, Newcastle University, Newcastle upon Tyne United Kingdom; ^3^ Department of Biosciences Durham University Durham United Kingdom; ^4^ Princess Al Jawhara Al‐Brahim Centre of Excellence in Research of Hereditary Disorders, King Abdulaziz University Saudi Arabia; ^5^ Clinical and Experimental Sciences, Faculty of Medicine University of Southampton Southampton United Kingdom; ^6^ Leeds Teaching Hospital NHS Leeds United Kingdom

**Keywords:** Age‐related macular degeneration, Complement factor H, Induced pluripotent stem cell, Retinal pigment epithelium

## Abstract

Age‐related macular degeneration (AMD) is the most common cause of blindness, accounting for 8.7% of all blindness globally. Vision loss is caused ultimately by apoptosis of the retinal pigment epithelium (RPE) and overlying photoreceptors. Treatments are evolving for the wet form of the disease; however, these do not exist for the dry form. Complement factor H polymorphism in exon 9 (Y402H) has shown a strong association with susceptibility to AMD resulting in complement activation, recruitment of phagocytes, RPE damage, and visual decline. We have derived and characterized induced pluripotent stem cell (iPSC) lines from two subjects without AMD and low‐risk genotype and two patients with advanced AMD and high‐risk genotype and generated RPE cells that show local secretion of several proteins involved in the complement pathway including factor H, factor I, and factor H‐like protein 1. The iPSC RPE cells derived from high‐risk patients mimic several key features of AMD including increased inflammation and cellular stress, accumulation of lipid droplets, impaired autophagy, and deposition of “drüsen”‐like deposits. The low‐ and high‐risk RPE cells respond differently to intermittent exposure to UV light, which leads to an improvement in cellular and functional phenotype only in the high‐risk AMD‐RPE cells. Taken together, our data indicate that the patient specific iPSC model provides a robust platform for understanding the role of complement activation in AMD, evaluating new therapies based on complement modulation and drug testing. Stem Cells *2017;35:2305–2320*

**Significance Statement**

Age‐related macular degeneration (AMD) is one of the most common forms of blindness. Drugs that treat wet AMD have been a major breakthrough; however, there is currently no treatment for the dry form. Some of the challenges associated with studying AMD are that the affected retinal tissue is difficult to obtain, there are no animal models that faithfully mimic the disease, and human trials are long and costly. This article reports creation of a disease model for AMD patients with the most common genetic risk factor for the disease. Strong evidence is presented to show that this model mimics the key features of AMD and can be used to test new therapies and to better understand the pathology of disease and the role of environmental, dietary, and lifestyle factors.

## Introduction

Age‐related macular degeneration (AMD) is the most common cause of blindness in the developed world, affecting one in three people by age 75 years, and is characterized by loss of central vision, affecting the macular area of the retina. It accounts for 50% of blind and partially sighted registration with an estimated prevalence of ∼600,000 significantly visually impaired people in the U.K. and over 8 million worldwide 1, 2, 3, 4. Approximately 70,000 new diagnoses are made every year in the U.K., and 13% of people aged over 80 years are affected by late stage AMD. The number of AMD affected people in the U.K. is expected to rise to 1.3 M by 2050 with healthcare costs rising to 16.4 billion during 2010–2020 5. Visual loss associated with AMD is caused by apoptosis of the retinal pigment epithelium (RPE) and overlying photoreceptors. AMD occurs in two forms: “dry” AMD where cellular debris, called drüsen accumulates between the RPE and Bruch's membrane (BrM), appearing as yellow specks on the retina. “Wet” AMD is usually characterized by aberrant blood vessel growth and encroachment from the choroid underneath the retina, although it can also originate from the inner retinal vasculature. Treatments are evolving for wet AMD including anti‐vascular endothelial growth factor (VEGF) treatments, photodynamic, and laser therapy 6, 7, 8; however, there are no effective treatments to prevent progression of the underlying disease process and advanced dry AMD.

AMD is a multifactorial progressive disease with a complex interaction between environmental, metabolic, hereditary factors, and chronic innate immune activation 9. A variety of alleles and haplotypes associated with early and late AMD have been identified from genome wide association studies (GWAS) 10, 11, 12, 13, but the precise roles of these genes and the mechanisms by which they increase disease risk are ill defined. One of the most significant genetic findings for AMD has been the complement factor H (*CFH*) polymorphisms. CFH protein functions by limiting the formation of C3 convertase of the complement system and by promoting the degradation of C3b to iC3b. Failure to control the activity of C3 convertase results in overproduction of C3b and C3a causing a shift in the complement cascade to its terminal lytic pathway. A significantly deleterious consequence of this is the formation of the anaphylotoxin, C5a, and the membrane attack complex (MAC) both of which deliver potent inflammatory signals. The T > C substitution in exon 9 (Y402H) of the *CFH* gene is strongly associated with susceptibility to AMD and has led to recognition of the importance of complement activation in AMD pathogenesis 10. There is now evidence from large case–control association studies to confirm association with a variety of other complement cascade genes including *CFHR1–3*, complement factor I (*CFI*), *CFB, C3,* and *C9* 10, 11. The polymorphisms within the 10q26 gene loci containing the *PlEKHA1/HTRA1/ARMS2* genes have also consistently demonstrated strong associations with AMD in GWAS 10, 12. In addition to data gathered from large genetic cohorts, biochemical and molecular studies have provided substantial evidence to support an important role for complement activation in AMD. This is illustrated by the presence of activators and regulators of the complement system in drüsen 14 and the increased expression of MAC proteins in choriocapillaris and BrM of aged individuals as well as those with the Y402H polymorphism 15, 16, 17.

The Y402H polymorphism can confer more than fivefold increase risk of developing AMD and is present in approximately 30% of people of European descent. Although factor H (FH) protein is synthesized by the choroid, it is not able to diffuse passively through BrM into the retina; however, its alternatively spliced, truncated form, named FH‐like protein 1 (FHL‐1), is able to do so 18. FHL‐1 retains all the necessary domains for complement regulation and binds to BrM through interactions with heparan sulphate 18, 19, 20. The Y402H polymorphism affects the ability of both FH and FHL‐1 to bind to heparan sulphate 21. Furthermore, FH and lipoproteins compete for binding to heparan sulphate in BrM 22; thus, it has been suggested that impaired binding of FH/FHL‐1 to heparan sulphate in individuals with Y402H polymorphism results in fewer binding sites for FH/FHL‐1, increased C3b depositions, lipoprotein accumulation, and failure to regulate complement activation, leading to recruitment of mononuclear phagocytes, RPE damage, and visual function decline.

Recent advances in the field of induced pluripotency have permitted generation of patient specific induced pluripotent stem cells (iPSCs), which have the ability to differentiate into cells of any tissue type including photoreceptors and RPE 23. The ability to produce large quantities of functional patient‐specific retinal cells from iPSCs offers an unparalleled chance to elucidate disease mechanisms and evaluate new therapeutic agents. Since the pathogenesis of AMD is largely unknown, creating a disease model using iPSC technology could be a valuable tool to address fundamental questions about disease biology as well as creating a biological tool to perform drug discovery and toxicity screening. The validity of this approach has been illustrated by two recent publications reporting derivation of iPSCs from AMD patients with *ARMS2/HTRA1* high‐risk genotypes displaying reduced superoxide dismutase 2 (SOD2) defense, rendering RPE more susceptible to oxidative damage 24, 25. We focused on derivation and characterization of iPSC from individuals homozygous for the low‐ and high‐risk *CFH* (Y402H) polymorphism. When compared with iPSC‐RPE derived from age matched control low‐risk individuals, the high‐risk iPSC‐RPE cells show a range of cellular, ultrastructural, and functional deficiencies that mimic several key features of AMD including increased inflammation, hallmarks of cellular stress, accumulation of lipid droplets and deposition of “drüsen”‐like deposits. Exposure to intermittent UV light elicited different responses from low‐ and high‐risk RPE cells and in the latter revealed an improvement in the cellular and ultrastructural features associated with AMD. Together, our data suggest that the patient‐specific iPSC disease modeling provides a robust tool to assess potential therapeutic agents to treat AMD before long expensive trials.

## Materials and Methods

### Human Donors

Written informed consent was obtained from each donor; all samples were obtained as part of a NHS research ethics committee approved biobank for fibroblasts from patients with retinal disease (ethics number 11/NE/0294) and based at the Institute of Genetic Medicine, Newcastle University, and adhered to the tenets set forth in the Declaration of Helsinki.

### iPSC Generation

Dermal fibroblasts were isolated from skin biopsies taken from patients with wet AMD and age‐matched donors with no clinical or genotypic indication of ocular disease (Supporting Information Fig. S1A, S1B). iPSCs were generated by Sendai viral transduction of the transcription factors *OCT4, SOX2, KLF4,* and *c‐MYC* (ThermoFisher, CytoTune2‐iPS Reprogramming Kit, Waltham, MA, https://www.thermofisher.com/uk/en/home.html) following manufacturer's instructions. iPSCs were maintained in defined conditions in mTeSR1 (StemCell Technologies, Vancouver, Canada, https://www.stemcell.com/) on growth factor‐reduced Matrigel (BD Biosciences, San Jose, CA, http://www.bdbiosciences.com/us/home).

### iPSC Characterization

The pluripotency of iPSC lines was confirmed with immunofluorescence, flow cytometry, and reverse transcriptase polymerase chain reaction (RT‐PCR). Cells were fixed with 4% paraformaldehyde for 15 minutes and stained with primary antibodies OCT4 and SSEA4 (Abcam, U.K., http://www.abcam.com/). Primary antibodies were detected using Alexa Fluor secondary antibodies (Supporting Information Fig. S4B). Nuclei were stained using 4′,6‐diamidino‐2‐phenylindole (DAPI). Images were captured using a fluorescence microscope (Leica Axiovert, Germany, http://www.leicabiosystems.com/). iPSC were assessed for their propensity to generate all three germ layers using primary antibodies against alpha‐fetoprotein (AFP), beta III Tubulin (TUJ1), and smooth muscle actin (SMA), primary antibodies were detected using Alexa Fluor secondary antibodies (3‐Germ Layer Immunocytochemistry Kit Invitrogen). RT‐PCR was used to detect mRNA transcripts of key pluripotency transcription factors, *NANOG, KLF4, c‐MYC,* and *SOX2* (Supporting Information Fig. S4C) and Sendai clearance (Supporting Information Fig. S5A). RNA was isolated from each iPSC line; RNA was isolated using a column biased method (Promega, http://www.promega.com/) and 1,000 ng of total RNA was reverse transcribed (GoScript Reverse Transcription System, Promega, http://www.promega.com/). Standard RT‐PCR was performed and PCR products electrophoresed on a 2% agarose gel.

Directly conjugated antibodies against NANOG (Cell Signalling, http://www.cellsignal.com/) and TRA‐1–60 (Millipore, U.K., http://www.emdmillipore.com/) (Supporting Information Fig. S4B) were used to detect the percentage population of pluripotent cells using BD fluorescence‐activated cell sorting (FACS) Canto II (BD Biosciences, San Jose, CA, http://www.bdbiosciences.com/us/home).

### Karyotyping

All cell lines were karyotyped using Illumina CytoSNP analysis and the BlueFuse Multi 4.3 software (Illumina, San Diego, CA, https://www.illumina.com/) according to standard protocols of the manufacturer.

### Generation of iPSC‐RPE

iPSC were allowed to reach 100% confluency, at which point the medium was switched from mTeSR1 to RPE differentiation medium (AdRPMI, ThermoFisher Scientific, B‐27 Supplement) (ThermoFisher Scientific, https://www.thermofisher.com/uk/en/home), 10% knockout serum replacement (ThermoFisher Scientific), 1% 100X GlutaMAX (ThermoFisher Scientific, https://www.thermofisher.com/uk/en/home), and 1% Penicillin–Streptomycin solution (ThermoFisher Scientific, https://www.thermofisher.com/uk/en/home). Cells were cultured for 16 days with medium replenished daily for the first 14 days. On day 16, the differentiation medium was supplemented with 2 µM Purmorphamine (StemCell Technologies, Vancouver, Canada, https://www.stemcell.com/) until day 21. Medium was replenished twice a week for the next 3–4 months. RPE patches were mechanically picked and placed in TryPLE (10X) (Invitrogen, https://www.thermofisher.com/uk/en/home/brands/invitrogen) for 30 minutes to dissociate the cells, agitated by gentle pipetting at 10, 20, and 30 minutes. Cells were sieved using a 100‐µm cell strainer and replated at 4.5 × 10^5^ cells per square centimeter on 24‐well plates or 0.33 cm^2^ PET hanging cell culture inserts (Merck Millipore; Billerica, http://www.emdmillipore.com/) coated with PLO/laminin (50 ng/μl) (Sigma‐Aldrich, https://www.sigmaaldrich.com/).

### Western Blotting

Supernatant was collected from apical portion of RPE cultures grown on transwell inserts incubated with Dulbecco's modified Eagle's medium (DMEM)/F12 for 4 days, soluble proteins were resolved on BioRad 4%–20% Tris‐glycine pre‐cast gels nonreduced (Bio‐Rad, http://www.bio-rad.com/) and transferred to nitrocellulose pre‐cut blotting membranes (ThermoFisher, http://www.thermofisher.com/uk/en/home). Blocking and antibody incubations were performed using 2% milk solutions. Membranes were incubated with primary antibody at 4°C overnight with gentle agitation. Primary antibodies were detected using applicable horseradish peroxidase (HRP)‐conjugated secondary antibodies (Supporting Information Fig. S4B). Detection of HRP‐labeled secondary antibody was performed with ECL SuperSignal Substrate (Pierce Biotechnology, http://www.thermofisher.com/uk/en/home/brands/thermo-scientific/pierce-protein-biology). Bands were identified by autoradiography with Carestream Kodak Biomax XAR film (Sigma‐Aldrich, http://www.sigmaaldrich.com/) and developed.

### DNA Extraction and Sequencing

DNA was extracted using a column biased method (Qiagen, Germany, https://www.qiagen.com/gb/), sequence tagged PCR was performed using 100 ng of DNA. Sanger sequencing was performed (GATC biotech, Germany, http://www.gatc-biotech.com/en/) and results were interpreted using finch TV http://jblseqdat.bioc.cam.ac.uk/gnmweb/download/soft/FinchTV_1.4/doc/ (Geospiza).

### Quantitative RT‐PCR

RNA was extracted from frozen cell pellets using ReliaPrep RNA Cell Miniprep System as per the manufactures instructions. RNA quantification was performed with a NanoDrop2000 spectrophotometer (ThermoFisher, http://www.thermofisher.com/uk/en/home). We ensured that the 260/280 ratio and concentration was between 1.7 and 2.1 and yields of >250 ng/μl. cDNA synthesis was performed using Promega, http://www.promega.com/ GoScript Reverse Transcription System as per the manufactures instructions. All experiments were performed using a QuantStudio 7 Flex Real‐Time PCR System (Applied Biosystems, U.K., http://www.thermofisher.com/uk/en/home/brands/applied-biosystems), using SYBR green reaction technology (Promega, U.K., http://www.promega.com/). Cycle parameters are as follows: 40 cycles of 95°C for 15 seconds and 60°C for 1 minute, finalizing with a melt curve stage. The Livak method (ΔΔ*C_t_*) was used 26. *C*_*t*_ results of the target genes were normalized to the *C*_*t*_ of the reference gene *GAPDH* (Δ*C_t_*); the Δ*C*_*t*_ obtained then normalized to the Δ*C*_*t*_ of the calibrator, yielding the (ΔΔ*C*_*t*_), finally the fold difference in expression was determined ( $2^{-\Delta \Delta C_{t}}$). A list of the primers used can be found in (Supporting Information Fig. S5B).

### RNA Sequencing

RNA was extracted using RNeasy Micro Kit (Qiagen, https://www.qiagen.com/) according to the manufactures instructions, from six‐cell culture inserts, three of each genotype. cDNA was generated using SMART‐Seq v4 Ultra Low Input RNA Kit (Clontech, https://www.clontech.com/). Sequencing was carried out on a NextSeq 500 (Illumina, https://www.illumina.com/). A 75‐bp paired‐end sequencing was carried out using a NextSeq 500 High Output v2 Kit (150 cycles) (Illumina, https://www.illumina.com/). RNA‐seq data were processed and analyzed to identify differentially expressed RNA. The quality of sequencing reads was first checked with FastQC (Version 0.11.2) 27. Poly‐N tails were trimmed off from reads with an in house Perl script. Low‐quality bases (Q < 30) and standard Illumina (Illumina, Inc. CA, https://www.illumina.com/) paired‐end sequencing adaptors on 3′ ends of reads were trimmed off using autoadapt (Version 0.2) and only those that were at least 20 bp in length after trimming were kept. The high quality reads were then mapped to the human reference genome hg38 with STAR (Version 2.5.0c) 28. Reads mapped to genes were then counted with HTSeq‐count (Version 0.6.1) 29 according to annotations from GENCODE, https://www.gencodegenes.org/ (Version 24) 30. Differentially expressed genes were identified with Bionconductor, https://www.bioconductor.org/ (Version 3.2) package DESeq2 (Version 1.10.1) 31. Genes differentially expressed in the 99.73 percentile, whereby genes that lie three SDs from the mean (μ‐3σ) were selected (more than or equal to fivefold change). This gene list was queried against the protein annotation through evolutionary relationship classification system to highlight disproportionally expressed pathways. RNA‐seq data are deposited into GEO (accession number GSE91087). A list of the overrepresented glycogenesis genes can be found in Table 6.

### Pigment Bleaching

Post fixation and before immunocytochemistry, RPE cells were bleached using a Melanin Bleach Kit (Polysciences, http://www.polysciences.com/) to remove pigmentation, as melanosomes can cause excessive autofluorescence. Pretreatment Solution A was added for 5 minutes at room temperature; the solution was removed and cells were washed two times with phosphate‐buffered saline (PBS). Pretreatment solution B was then added for 1–3 minutes until pigmentation was removed. This solution was removed and cells were washed again with PBS.

### Phagocytosis of Rod Outer Segments

Bovine POS was obtained from InVision BioResources (Seattle, http://www.invisionbio.com/). Before performing the assay, POS were FITC labeled using the following procedure. The POS were centrifuged at 4,500*g* for 4 minutes. They were then resuspended in AdRPMI, 10% fetal bovine serum (FBS) with 0.4 mg/ml FITC (Sigma, https://www.sigmaaldrich.com/) and incubated for 1 hour at room temperature protected from light. This was followed by another centrifugation for 4 minutes at 4,500*g*. POS was washed three times with PBS and then resuspended in 2.5% sucrose (Sigma, https://www.sigmaaldrich.com/) in PBS and stored in −80°C until further use. For phagocytosis experiments, normal RPE cell medium was changed to the POS medium (AdRPMI, B27, 10% FBS). Once thawed, POS were resuspended in POS medium. Approximately 1 × 10^6^ FITC‐labeled POS were added per cell culture insert for 4 hours at 37°C. In parallel, negative control experiments were performed where cells were kept for the same duration but at 4°C. The incubations were followed by two cell washes with PBS. Cells were then dissociated with TrypLE Select Enzyme (10X) (ThermoFisher Scientific, https://www.thermofisher.com/uk/en/home) and washed. They were then resuspended in 2% FBS solution in PBS with the addition of DRAQ5 (1:400, BioStatus; Shepshed, U.K., http://www.biostatus.com/) for 5 minutes. Extracellular fluorescence was quenched with 0.2% trypan blue stain (ThermoFisher Scientific, https://www.thermofisher.com/uk/en/home) for 10 minutes. Cells were then washed at least three times with PBS and resuspended in 2% FBS solution in PBS. Cells were analyzed on a BD™ LSR II flow cytometer (BD Biosciences; Franklin Lakes, http://www.bdbiosciences.com/us), collecting at least 10,000 events per sample. The data were analyzed on BD FACSDiva software (BD Biosciences, http://www.bdbiosciences.com/us) using following equation

$$\%=\left(\frac{\text{FITC}\;\text{positive}}{\text{Total}\;\text{number}\;\text{of}\;\text{cells}}\right)\times 100$$

### Immunofluorescence

Cells were washed once with PBS, followed by fixation with 4% paraformaldehyde for 15 minutes at room temperature. Cells were then washed once with PBS, followed by blocking in 0.25% Triton‐X‐100 (Sigma, https://www.sigmaaldrich.com/) and 5% NGS (Thermo Scientific, https://www.thermofisher.com/uk/en/home) in PBS for 1 hour. This solution was removed and replaced with antibody diluent (0.25% Triton‐X‐100, 1% bovine serum albumin in PBS) with applicable antibody dilution; [Anti‐ZO1, rabbit (Invitrogen; dilution 1:200, https://www.thermofisher.com/uk/en/home/brands/invitrogen), Anti‐C5b‐9 (Dako 1:200), Anti‐apolipoprotein E (APOE) (Merck Millipore 1:1,000, http://www.emdmillipore.com/), Anti‐LC3 (Cell Signaling 1:250, http://www.cellsignal.com/), Anti‐p62 (1:500)] (Supporting Information Fig. S4B) at 4°C overnight. Cells were washed three times in PBS followed by incubation with secondary antibodies [Cy™3 AffiniPure Goat Anti‐Rabbit IgG (Jackson ImmunoResearch, http://www.jacksonimmuno.com/ (West Grove); dilution 1:1,000), Anti‐mouse IgG–FITC antibody (Sigma; dilution 1:1,000), Donkey anti‐Goat 488 (1:500, Abcam, http://www.abcam.com/), Donkey anti mouse 647 (Abcam 1:500, http://www.abcam.com/)] for 1 hour at room temperature. Cells were washed as stated previously and mounted in Vecta sheild (Vector Labs, https://vectorlabs.com/uk/) with Hoechst 33342 (1:1,000, Thermo Scientific, https://www.thermofisher.com/uk/en/home), TO‐PRO‐3 (1:1,000, Thermo Scientific) or DAPI (Sysmex, 1:1,000, https://www.sysmex-partec.com). C5b‐9 and APOE signals were detected using a Nikon A1R confocal (resonant, invert) (Nikon, Japan, https://www.nikoninstruments.com/Applications/Life-Sciences), 45–50 µm optimally sampled. Image processing was performed using Huygens Essential (Germany, https://svi.nl/Huygens-Essential) co‐localization with the threshold set to 1%.

### In Vitro and In Vivo 3 Germ Layer Differentiation

In vitro iPSCs were spontaneously differentiated to allow the emergence of cell types representative of the three embryonic germ layers. iPSCs were allowed to reach 80% confluency after which the media were switched to DMEM/F12 (ThermoFisher Scientific, https://www.thermofisher.com/uk/en/home), 20% FBS (ThermoFisher Scientific, https://www.thermofisher.com/uk/en/home), 1% Penicillin–Streptomycin solution (ThermoFisher Scientific), and 1% MEM non‐essential amino acids solution (ThermoFisher Scientific, https://www.thermofisher.com/uk/en/home). Medium was replaced daily for 3 weeks. The 3‐Germ Layer Immunocytochemistry Kit (ThermoFisher Scientific, https://www.thermofisher.com/uk/en/home) was used to detect cells positive for markers of mesoderm, endoderm, and ectoderm. Briefly, media were removed from cellular monolayers, followed by a 15‐minute incubation with fixative solution. Fixative was removed and permeabilization solution was added for 15 minutes. This was then removed and replaced with blocking solution for 1 hour, after which the applicable antibody was added: SMA (mesoderm), AFP (endoderm), or Anti‐TUJ1 (ectoderm). Cells were washed three times for 3 minutes in wash buffer. Secondary antibody was then added (Supporting Information Fig. S4B) for 1 hour, after which the cells were washed as stated previously and 1–2 drops/ml of NucBlue was added. Plates were stored at 4°C before imaging on a Zeiss Axioplan microscope, https://www.micro-shop.zeiss.com. All incubations occurred at room temperature unless otherwise stated.

### Teratoma Formation in Immunodeficient Mice

All procedures were approved and conformed to institutional guidelines. Approximately 1 × 10^6^ iPSCs were injected subcutaneously into the right flank of adult non‐obese diabetic/severe combined immunodeficient mice. All cells were cotransplanted in a 50‐µl Matrigel carrier, (BD Biosciences, http://www.bdbiosciences.com/us/home) to enhance teratoma formation. Mice were killed after 70–90 days, and teratoma tissues were extracted. Teratoma material for histological analysis was fixed in Bouins fixative [70% saturated picric acid (Sigma, https://www.sigmaaldrich.com/); 25% formaldehyde (37%/40%, Sigma) and 5% glacial acetic acid (Sigma, https://www.sigmaaldrich.com/)] overnight. Tissues were processed, then sectioned to 6 μm, and then counterstained with either H&E or Massons trichrome stain. Sections were assessed using bright field microscopy on an Axio Imager (Lecia, Germany, http://www.leicabiosystems.com/).

### Trans‐Epithelial Resistance

Trans‐epithelial resistance (TER) was measured with a Millicell ERS‐2 Voltohmmeter (Merck Millipore, http://www.emdmillipore.com/). First, the electrical resistance of a blank cell culture inserts with media in both apical and basal compartments was measured, after which inserts with cells were measured. A minimum of two repeated measurements were made of each insert. TER was calculated using the following formula:

$$\text{TER}\left(\Omega {\text{cm}}^{2}\right)=\left(\frac{\text{average}\;\text{blank}}{\text{average}\;\text{sample}}\right)\times \text{area}.$$

### Transmission Electron Microscopy

Cells were fixed with 2% glutaraldehyde and kept at 4°C. Transmission electron microscopy (TEM) including all the cell processing was performed at Newcastle University Electron Microscopy Research Services. Ultrathin sections were stained with heavy metal salts (uranyl acetate and lead citrate) and imaged on a Philips CM100 TEM.

### Statistical Analysis

Shapiro‐Wilk test was used to determine normality, for normally distributed data sets, one‐way analysis of variance (ANOVA) followed by Tukey's post hoc test was used to analyze intergroup differences between samples. Two‐way ANOVA was used to compare between samples and treatment groups. For non‐normally distributed data, Wilcoxon matched‐pairs signed rank test was utilized in matched samples, while Mann Whitney test were used all other times. GraphPad Prism 7.0 (San Diego, CA, https://www.graphpad.com/scientific-software/prism/) was used to perform all statistical analyses. Data are presented as mean ± SD and a confidence interval of 95% was set, and *p* ≤ .05 was considered statistically significant.

## Results

### Generation of iPSCs from High‐Risk AMD Donors and Unaffected Controls

To investigate how the Y402H polymorphism in *CFH* leads to the pathology associated with AMD, DNA was extracted from donor cell fibroblasts and sequenced to detect single nucleotide polymorphisms in the *CFH, HTRA1*, and *ARMS2* genes (Supporting Information Fig. S1A). The two homozygous low‐risk donors were selected on the basis of low‐risk for all three single nucleotide polymorphisms (SNPs) rs11200638 (*HTRA1*), rs1061170 (*CFH*), and rs10490924 (*ARMS2*) and no clinical manifestation of AMD. The high‐risk donors were specifically selected as having advanced AMD with unilateral wet AMD and reticular pseudo‐drüsen (a known high‐risk feature for both types of advanced AMD) in their fellow eyes (Supporting Information Fig. S1B, S1C′) and high‐risk SNP for *CFH* and low‐risk *HTRA1* and *ARMS2*. The high‐risk *CFH* in combination with low‐risk *HTRA1* polymorphism has been consistently associated with central drüsen formation in the older age group 32.

iPSCs were generated from dermal fibroblasts using nonintegrative Sendai viral vectors expressing Yamanaka reprogramming transgenes. Between 20 and 30 clones were generated from each donor. At least three clones from each individual were expanded, adapted to feeder free culture conditions and thoroughly characterized using well established tests of pluripotency including expression of markers by immunocytochemistry (SSEA4 and OCT4; Supporting Information Fig. S2A), RT‐PCR (*NANOG, KLF4, C‐MYC*, and *SOX2*) (Supporting Information Fig. S2B), flow cytometry (SSEA4 and OCT4; Supporting Information Fig. S2C), and has the ability to differentiate into all three germ layers in vitro (SMA, TUJ1, and AFP; Supporting Information Fig. S2D) and in vivo (Supporting Information Fig. S2E), clearance of Sendai Transgenes (Supporting Information Fig. S2F), and genetic identity to parent fibroblasts (Supporting Information Fig. S2G). One clone from each patient was further selected for differentiation studies. CytoSNP analysis indicated no chromosomal rearrangements, losses, or duplications. Our RT‐PCR analysis also indicated that *CFH and FHL‐1* are not expressed in iPSCs (Supporting Information Fig. S3A).

### Establishing iPSC‐RPE from High‐Risk AMD Patients and Unaffected Controls

**Figure 1:**
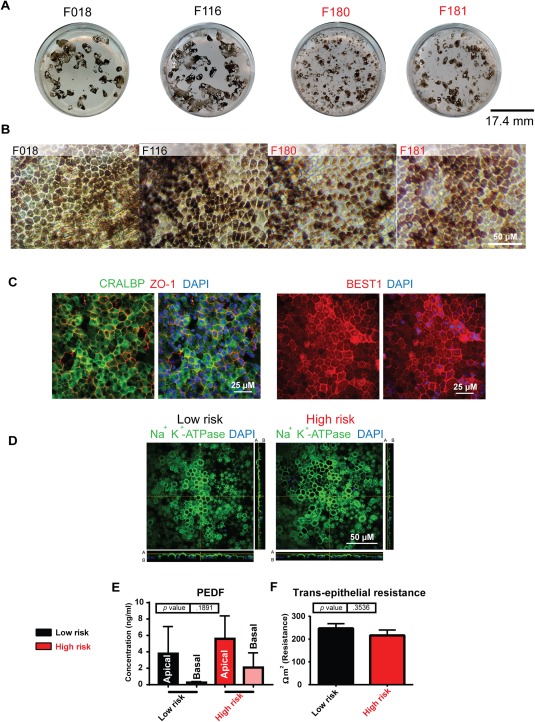
Derivation of age‐related macular degeneration (AMD)‐induced pluripotent stem cell (iPSC) retinal pigment epithelium (RPE). **(A)**: Light microscopy images of iPSC‐RPE cells derived from low‐risk donors (F018 and F116:402Y) and high‐risk AMD patients (F180 and F181:402H). Scale bar = 50 µm. **(B)**: Flatbed scanned images of a representative well from six‐well plates, pigmented patches are clearly visible. Scale bar = 17.4 mm. **(C)**: CRALBP/ZO‐1 and BEST1 immunostaining of representative iPSC‐derived RPE cells, Scale bar = 25 µm. **(D)**: Immunofluorescence representative images of iPSC‐RPE and orthogonal images along *x*‐ and *y*‐axis (green, Na^+^ K^+^‐ATPase) (blue, 4′,6‐diamidino‐2‐phenylindole) Scale bar = 50 µm, A, apical; B, basal. **(E)**: Pigment epithelium‐derived factor enzyme‐linked immunosorbent assay of apical and basal supernatant, concentration nanogram per milliliter. **(F)**: Trans‐epithelial resistance data shown as Ωcm^2^ (resistance). Abbreviations: DAPI, 4′,6‐diamidino‐2‐phenylindole; PEDF, pigment epithelium‐derived factor.

iPSCs were differentiated to RPE using a defined serum and feeder free protocol described in the Materials and Methods section. The RPE patches were mechanically isolated and expanded on laminin coated transwell inserts or tissue culture plates. Hexagonal cells with pigmentation both visible macro‐ and microscopically (Fig. 1A, 1B), which expressed the putative RPE cell markers ZO‐1, CRALBP, and BEST1 (Fig. 1C). Polarity in the RPE cells is important for their physiological function, we checked the presence of Na^+^ K^+^‐ATPase in both low‐ and high‐risk iPSC‐RPE cells and showed apical localization in both (Fig. 1D). iPSC‐RPE cells secreted pigment epithelium‐derived factor also known as serpin F1 in a physiologically similar fashion to adult RPE 33 (Fig. 1E) all cultures. RPE cells form a tight barrier in the retina which can be measured by TER. We observed no significant differences in TER between RPE derived from high‐ or low‐risk AMD individuals (Fig. 1F). Phagocytosis assays also indicated that iPSC‐RPE were able to phagocytose bovine rod outer segments with no differences observed between low‐ and high‐risk AMD donors (data not shown).

### Expression of CFH, FH‐Like Protein 1, FI, and C3b in iPSC‐RPE Cells

**Figure 2:**
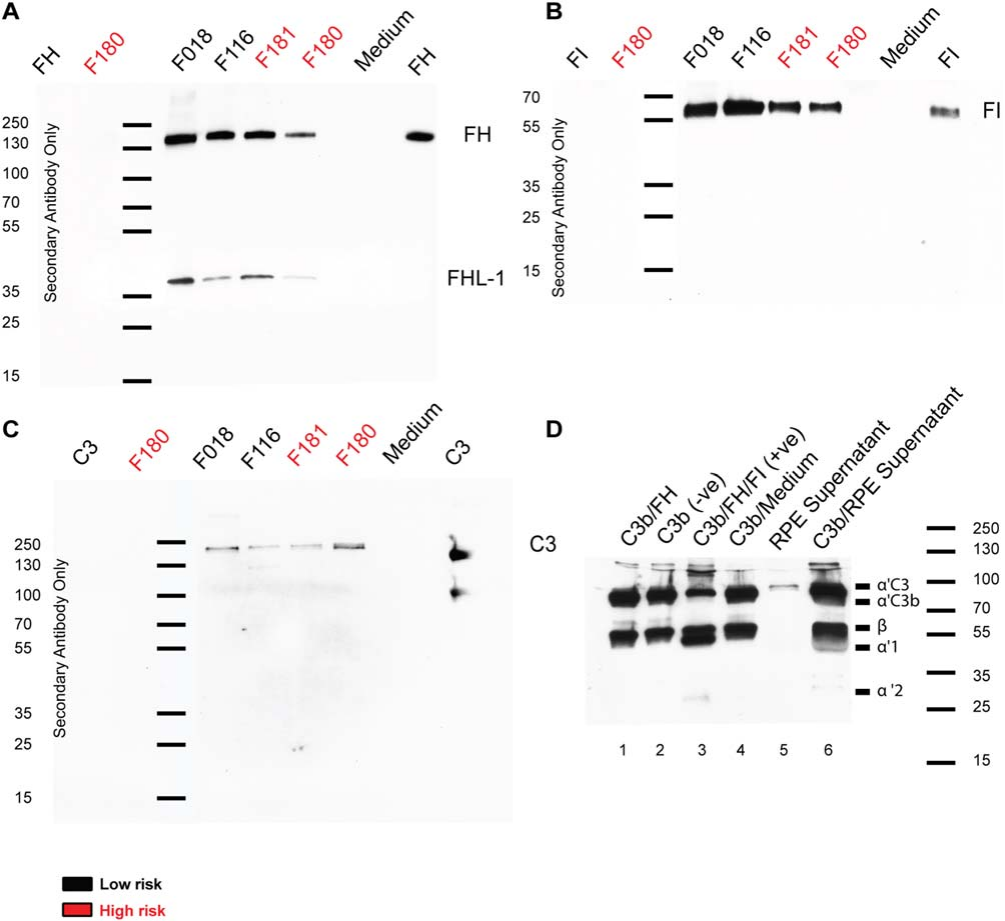
Expression and secretion of complement proteins by induced pluripotent stem cell (iPSC)–retinal pigment epithelium (RPE) by Western blotting. **(A)**: Western blot of protein excreted into the medium by RPE cells, anti‐factor H (FH) antibody (ab) shows a band visible at 150 kDa and factor H‐like protein‐1 visible at 37 kDa. **(B)**: Anti‐factor I (FI) ab indicates a band at 65 kDa. **(C)**: C3 is visible at 187 kDa, while C3b is also visible in the positive control at 90 kDa. **(D)**: Western blot of a fluid phase tripartite cofactor assay probed with anti‐C3 ab. Supernatant from age‐related macular degeneration patient induced pluripotent stem cell (iPSC)‐RPE cells cultured for 48 hours in serum free media was incubated with purified C3b to test the activity of FI and FH previously demonstrated to be secreted by these cells (A, B) (lane 6). Activity was confirmed by the generation of a 67‐kDa α′1 chain and a 40‐kDa α′2 chain. It is also observed that these cells had secreted C3 into the supernatant (C) (lane 5). The serum free media alone had no effect on C3 (lane 4). C3b/FH combined had minimal cleavage (lane 1) and C3b alone was not cleaved (lane 2). C3b/FH/FI combined was able to cleave C3b to the 67‐kDa α′1 chain and a 40‐kDa α′2 chain in the positive control (lane 3). Abbreviations: FH, factor H; FHL‐1, factor H‐like protein 1; FI, factor I; RPE, retinal pigment epithelium.

**Figure 3:**
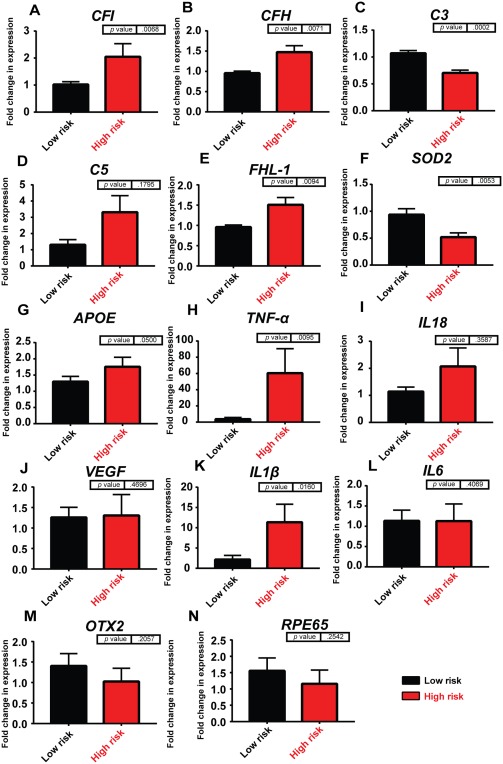
Gene expression analysis in low‐ and high‐risk age‐related macular degeneration induced pluripotent stem cell (iPSC)‐retinal pigment epithelium (RPE). Data are presented as mean ± SEM, *n* = 3. Abbreviations: APOE, apolipoprotein E; CFH, complement factor H; CFI, complement factor I; FHL‐1, factor H‐like protein 1; IL, interleukin; OTX, orthodenticle homeobox 2; RPE, retinal pigment epithelium; SOD, superoxide dismutase; TNF, tumor necrosis factor; VEGF, vascular endothelial growth factor.

Expression of FH/FHL‐1 (Fig. 2A), factor I (FI) (Fig. 2B), and C3 proteins (Fig. 2C) were detected in RPE conditioned supernatants derived from both high‐ and low‐risk cultures, indicating that iPSC‐RPE cells secrete the main component and regulators of the alternative complement pathway. Importantly, the excreted proteins FH and FI were shown to be functional, indicated by the breakdown of C3b to iC3b (Fig. 2D). *CFH, FHL‐1*, *CFI*, and *C3* expression were also confirmed at the mRNA level utilizing quantitative RT‐PCR (Fig. 3A–3E). *CFI, CFH*, and *FHL‐1* were all upregulated in high‐risk iPSC‐RPE cells (Fig. 3A–3C*)*, a trend also observed in iPSC‐RPE derived from HTRA1/ARMS2 risk genotypes 25. Interestingly, C3 was downregulated in high‐risk iPSC‐RPE, while C5 showed no significant difference (Fig. 3C, 3D). Together, these results add to the evidence for local complement synthesis in the eye as documented previously in the literature 34, 35.

### Gene Expression Profiles of Cytokines in AMD and Control iPSC‐RPE

High‐risk iPSC‐RPE had reduced gene expression of mitochondrial *SOD2* (Fig. 3F), which acts to transform superoxide, a toxic by‐product of oxidative phosphorylation, into less harmful hydrogen peroxide and diatomic oxygen. It has also been reported that ARMS2/HTRA1 polymorphism leads to compromized SOD2 response 23, while knockout of *SOD2* in mice is used as an early model of AMD 36. *APOE*, a transporter of lipoproteins and fat‐soluble vitamins, and tumor necrosis factor (*TNF*)*‐α*, a cytokine involved in systemic inflammation and implicated in downregulation of orthodenticle homeobox 2 (*OTX2*) 37, showed increased expression in high‐risk donors (Fig. 3G, 3H); however, interleukin‐18 (*IL18*), a cytokine that can suppress *VEGF* expression and has been associated with AMD 38 showed no significant difference between genotypes (Fig. 3I). No significant changes between genotypes were observed for *VEGF* expression (Fig. 3J). IL1β has a broad spectrum of mediation in cellular function and has been implicated as an effector of the inflammatory response 39. We noted a significant difference between genotypes with high‐risk donors expressing *IL1β* at a higher level than low‐risk RPE (Fig. 3K). Elevated levels of *IL‐6* are found in the vitreal fluid of AMD patients and have been used as predictors of AMD progression 40; however, we did not detect any difference in IL6 between genotypes suggesting that the release of this cytokine is likely from another source such as microglia (Fig. 3L). *OTX2* controls essential, homeostatic RPE genes. There was a slight decrease (although not significant) in *OTX2* in high‐risk donors, perhaps linked to *TNF‐α* expression as stated previously (Fig. 3M). *RPE65* expression remained constant in both genotypes (Fig. 3N).

To further probe into the differences between high‐ and low‐risk RPE cells, we performed RNA‐seq studies which identified 41 genes residing in the 0.3 percentile and 99.7 percentile, equating to a −5.532‐fold change or greater and 5.66 or greater, respectively (Supporting Information Fig. S3C). This analysis revealed the upregulated expression of *CGA* (Glycoprotein hormones α‐chain) in RPE cells derived from high‐risk donors (Supporting Information Fig. S3C). Currently, there is no documented expression of CGA in RPE cells; however, the expression of receptor (GNRHR, Gonadotropin‐releasing hormone receptor) has been detected in retinal tissues 41. Recoverin (*RCVRN)* expression, which marks photoreceptor precursors, was also upregulated in high‐risk donors (Supporting Information Fig. S3C). It is of interest to note that expression of *RCVRN* has been observed in RPE cells which were induced to transdifferentiate to photoreceptors via overexpression of *Neurogenin* 1 or 3 40. RPE cells undergoing this transition have been noted to retain pigmentation while displaying an elongated cell body and *RCVRN* expression in aPVMD2‐ngn1 mouse 42, 43. It is currently unclear whether increased *RCVRN* expression in high‐risk AMD‐RPE is related to cell fate changes or to an impaired wound healing response and epithelial to mesenchymal transition which has already been reported in AMD‐RPE 44. One family of genes was disproportionately represented in the low‐risk donor including members of the Wnt and Cadherin signaling pathways [Protocadherin gamma‐A3 (PCDHGA3), Protocadherin beta‐8 (PCDHB8), Protocadherin gamma‐A6 (PCDHGA6), and Putative protocadherin beta‐18 (PCDHB18P)], implying that low‐risk donors may find it easier to form cell–cell junctions when compared with high‐risk RPE; thus, corroborating previously published data showing disrupted cell to cell junctions and induction of AMD‐associated pathological changes in light exposed RPE cells 45.

When all genes with >1.5‐fold expression changes between the high‐ and low‐risk iPSC RPE were analyzed using Enrichr, (OMIM disease) 46, macular degeneration, diabetes mellitus type 2, and protein glycosylation disorder diseases were found to be overrepresented in the RNA‐seq dataset (*p = .05475*, Supporting Information Fig. S Fig. S3 B). Glycosyltransferases are responsible for post‐translational glycol modification of proteins, and this is considered a location specific modification as the enzymes required are normally compartmentalized. Glycosylation status is suggested to be important for efficient transport/diffusion of FHL‐1 though BrM; FHL‐1 is normally nonglycosylated and passes easily 47, while glycosylated CFH does not. Interfering with this status could be detrimental to location and diffusion characteristics. Additionally, advanced glycosylation end products are a classical indication of an aged RPE cell layer 48. Together, these data suggest that the in vitro iPSC‐RPE model we have created mimics the disease at the molecular level.

### C5b‐9 Deposition and APOE Colocalization in AMD iPSC‐RPE

**Figure 4:**
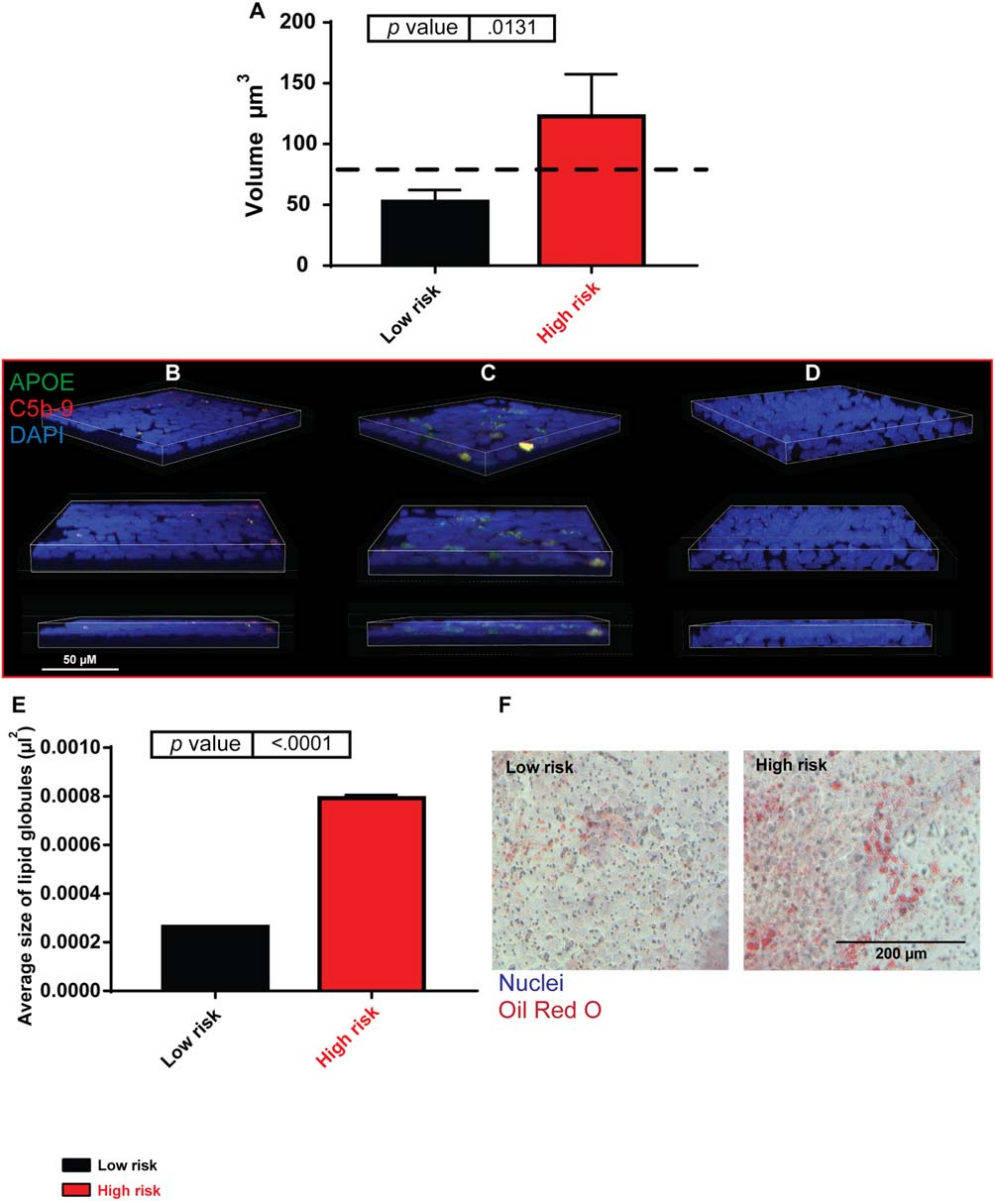
Drüsen‐like deposits form in high‐risk induced pluripotent stem cell (iPSC)‐retinal pigment epithelium (RPE). **(A)**: Volume of apolipoprotein E (APOE) and C5b‐9 coexpressing deposits in low‐ and high‐risk iPSC‐RPE, dashed black line represents clinically significant drüsen size. High‐risk donor iPSC‐RPE cells accumulate larger deposits than low‐risk, *n* = 3. **(B)**: Low‐risk iPSC‐RPE example of F116; blue, 4′,6‐diamidino‐2‐phenylindole (DAPI); red, C5b‐9; and green, APOE. Scale bar = 50 µm. **(C)**: High‐risk iPSC‐RPE example of F181; blue, DAPI; red, C5b‐9; and green, APOE. Scale bar = 50 µm. **(D)**: Secondary antibody only control; blue, DAPI, red, C5b‐9; and green, APOE. Scale bar = 50 µm. **(E)**: Oil red O staining, high‐risk donor iPSC‐RPE contained larger lipid globules than low‐risk donors. **(F)**: Examples of low‐ and high‐risk donor oil red O staining. Scale bar = 200 µm. Abbreviations: APOE, apolipoprotein E; DAPI, 4′,6‐diamidino‐2‐phenylindole.

Many investigations have described the proteomic and lipid composition of drüsen 49. APOE is ubiquitously associated with drüsen formation and shown to comprise 36% of all proteins found extracellularly 49. The terminal complement complex (C5b‐C6‐C7‐C8‐C9_n_, [C5b‐9]) is comprised of five proteins, C5b, C6, C7, and C8, with the fifth, C9 forming a transmembrane ring structure. We found the presence of aggregates that either contained ApoE, C5b‐9, or both proteins in low‐ and high‐risk iPSC‐RPE; however, the size of deposits containing both ApoE and C5b 9 was larger in the high‐risk RPE (Fig. 4A–4D). Significantly, larger lipid globules were also detected in high‐risk donors compared with low‐risk (Fig. 4E, 4F).

### Ultrastructural Changes to AMD iPSC‐RPE

**Figure 5:**
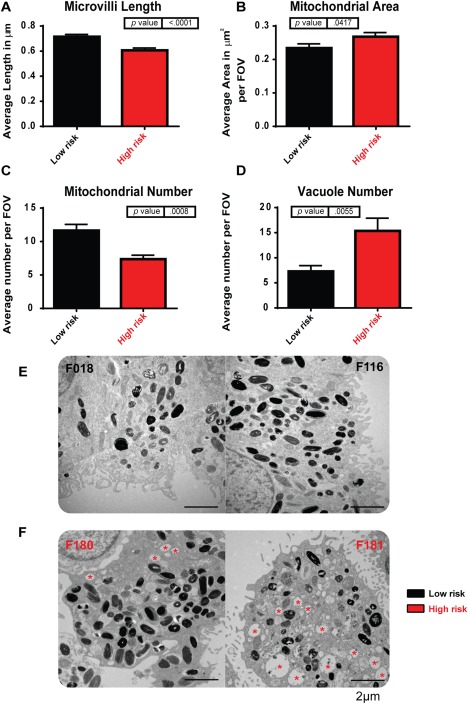
High‐risk induced pluripotent stem cell (iPSC)‐retinal pigment epithelium (RPE) shows ultrastructural changes in transmission electron microscopy analysis. **(A)**: Microvilli length (calculated per field of view: 100 µm^2^) is decreased in high‐risk donor iPSC‐RPE, *n* = 3. **(B)**: Mitochondrial area (calculated per field of view: 100 µm^2^) was increased in high‐risk donor iPSC‐RPE, *p* = .0417, *n* = 3. **(C)**: Mitochondrial number (calculated per field of view: 100 µm^2^) was decreased in high‐risk donor iPSC‐RPE, *p* = .0008, *n* = 3. **(D)**: The number of vacuole structures (calculated per field of view: 100 µm^2^) was greatly increased in high‐risk donor iPSC‐RPE, *p* = .0055, *n* = 3. **(E)**: Examples of low‐risk iPSC‐RPE cells: left hand side, F018; right hand side, F116; Scale bar = 2 µm. **(F)**: Examples of high‐risk iPSC‐RPE cells: left hand side, F180; right hand side, F181; red asterisk indicates vacuoles. Scale bar = 2 µm. Abbreviation: FOV, field of view.

TEM showed that the length of microvilli was reduced in RPE derived from the high‐risk donors (Fig. 5A). The mitochondrial number also decreased (Fig. 5C); however, the area covered by them was slightly larger in high‐risk donors (Fig. 5B) suggestive of fewer but larger mitochondria which could be the result of age related mitochondrial dysfunction or stress 50. Long range PCR assays indicated the absence of mitochondrial DNA deletions in the fibroblasts and RPE derived from both low‐ and high‐risk individuals (data not shown). We also observed the formation of asymmetric vacuoles (marked with‐red stars) almost exclusively in RPE generated from high‐risk donors (Fig. 5D–5F). These vacuoles, which are indicative of “adaptive survival” in response to environmental or oxidative stress, have also been observed in a *SOD2* knockdown mouse model of early AMD 34. They have the potential to lead to vacuolation‐mediated cell death; however, our flow cytometric analysis did not indicate significant changes in apoptosis between low‐ and high‐risk AMD iPSC‐RPE (data not shown).

### Autophagy Is Upregulated in High‐Risk AMD iPSC‐RPE Cells

**Figure 6:**
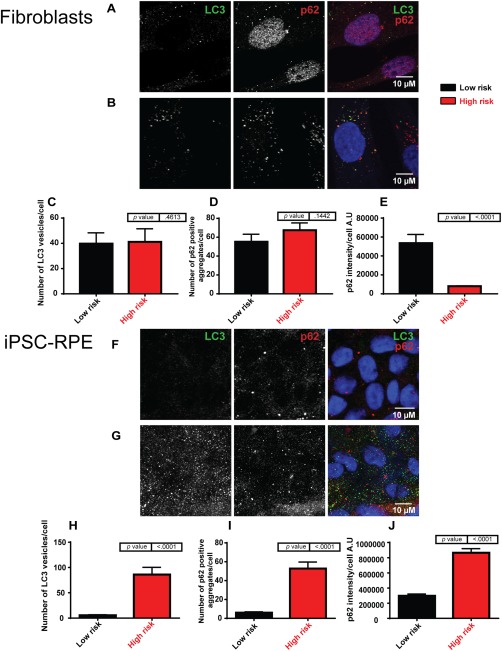
Autophagy is blocked in high‐risk induced pluripotent stem cell (iPSC)‐retinal pigment epithelium (RPE) cells but not in high‐risk dermal fibroblasts. **(A)**: Examples for fluorescence staining of low‐risk fibroblasts: blue, 4′,6‐diamidino‐2‐phenylindole (DAPI); green, LC3; and red, p62. Scale bar = 10 µm. **(B)**: Examples for fluorescence staining of high‐risk fibroblasts: blue, DAPI; green, LC3; and red, p62. Scale bar = 10 µm. **(C)**: The number of LC3 vesicles per cell did not differ between genotypes in iPSC‐RPE cells. **(D)**: Number of p62‐positive aggregates per cell was not affected by genotype **(E)**:. The intensity of p62 decreases in high‐risk age‐related macular degeneration iPSC‐RPE cells. **(F)**: Examples for fluorescence staining of low‐risk iPSC‐RPE: blue, TO‐PRO‐3; green, LC3; and red, p62. Scale bar = 10 µm. **(G)**: Examples for fluorescence staining of high‐risk iPSC‐RPE: blue, TO‐PRO‐3; green, LC3; and red, p62. Scale bar = 10 µm. **(H)**: LC3 aggregates were significantly increased in high‐risk iPSC‐RPE **(I)** p62 aggregates per cell were significantly increased in high‐risk **(J)** p62 intensity also increased. Abbreviations: iPSC, induced pluripotent stem cell; RPE, retinal pigment epithelium.

Due to the increased lipid build‐up and ultrastructural changes, we suspected that autophagy may have a role in AMD pathogenesis. It has also been documented previously that dysregulated autophagy may sensitize RPE cells to oxidative stress 51. Autophagy is associated with intra/inter cellular waste removal and is upregulated during nutrient starvation and general stress response. In donor fibroblasts, no difference in expression of two key autophagy markers, LC3 puncta and p62 aggregates was observed between low‐ and high‐risk donors (Fig. 6A–6D); however, p62 intensity was higher in low‐risk donors (Fig. 6E) and localized to the nuclei. In the corresponding iPSC‐RPE, LC3 puncta and p62 aggregates were greatly upregulated in high‐risk RPE (Fig. 6F–6I) along with the intensity of p62, which was also increased (Fig. 6J), which potentially suggests a block in autophagy in high‐risk RPE‐iPSC.

### Response of Low‐ and High‐Risk AMD‐RPE Cells to UV Exposure

UV can induce the generation of reactive oxygen species (ROS) derived from diatomic oxygen (O_2_), superoxide anion (O_2_‐), hydroxyl, and peroxyl radicals, resulting in DNA damage. The retina is highly susceptible to photochemical damage due to continuous light and UV exposure. This photochemical induction is exacerbated by the retinal oxygen tension (70 mmHg), which is higher than many other tissues, thereby increasing the probability of ROS formation. Although the relationship between UV light exposure and AMD is unclear, epidemiological evidence indicates an association between the severity of light exposure and the occurrence of AMD 52. Light in the visible UV spectrum (441 nm) is deleterious for RPE cells, being the most energetic radiation reaching the macula and causes photo‐oxidation generating reactive photoproducts including *N*‐retinylidene‐*N*‐retinylethanolamine (A2E), DNA oxidation, and cell apoptosis 53, 54. Drüsen and outer segments are composed largely of lipids (polyunsaturated fatty acids) and are particularly vulnerable to photo‐oxidation leading to a chain reaction mechanism of lipid peroxidation and peroxide organic free radical production 55.

**Figure 7:**
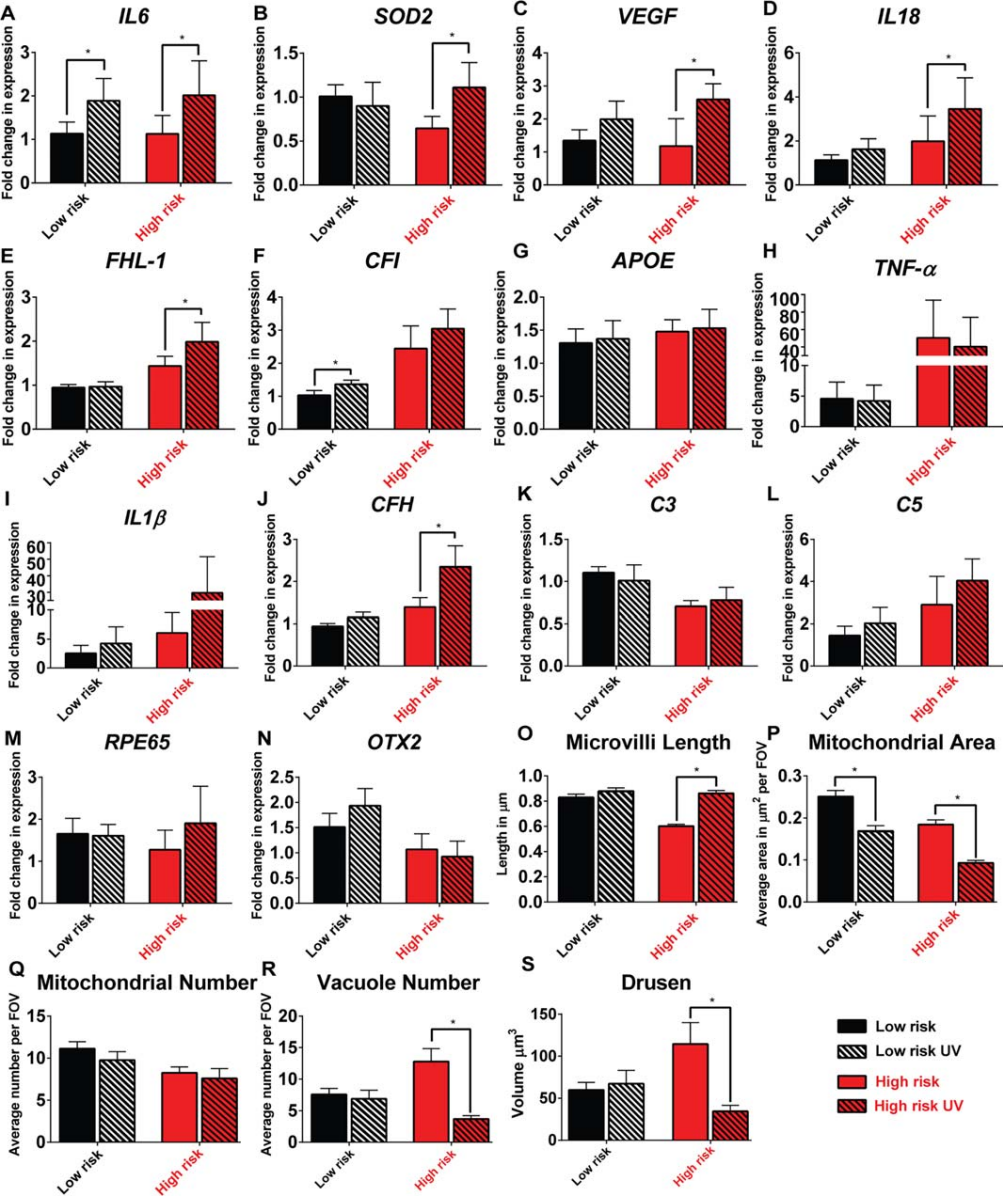
The response of low‐ and high‐risk retinal pigment epithelium (RPE) to intermittent UV exposure. **(A–N)**: Quantitative reverse transcriptase polymerase chain reaction expression data shown as fold change in relation to low‐risk control; data are presented as mean ± SEM, *n* = 3, *, *p <* .05. [Correction made here after initial online publication.] **(O)**: Average length of microvilli per field of view (100 µm^2^), UV exposure increased microvilli length in high‐risk induced pluripotent stem cell (iPSC)‐RPE cells, *p* < .0001. **(P)**: Average mitochondrial area per field of view (100 µm^2^), UV exposure decreased mitochondrial area in both low‐ and high‐risk iPSC‐RPE (*p =* .0003 and *p* = .0002). **(Q)**: Average mitochondrial number per field of view (100 µm^2^), UV exposure did not influence the average number of mitochondria (*p* = .9789). **(R)**: Average vacuole number (calculated per field of view: 100 µm^2^), UV exposure decreased the number of vacuoles in high‐risk cells, *p* = .0038. **(S)**: Drüsen area, UV exposure decreased the deposition of C5b‐9 and apolipoprotein E in high‐risk iPSC‐RPE cells, *, *p* = .0002. Abbreviations: APOE, apolipoprotein E; CFI, complement factor I; FHL‐1, factor H‐like protein 1; FOV, field of view; IL, interleukin; OTX, orthodenticle homeobox 2; RPE, retinal pigment epithelium; SOD, superoxide dismutase; TNF, tumor necrosis factor; VEGF, vascular endothelial growth factor.

To investigate whether UV exposure acts to exacerbate the gene expression, functional or structural defects observed in RPE cells derived from AMD patients, we exposed iPSC‐RPE cells continuously to 0.0045 mW/cm^2^ of 390–410 nm light for 1 hour each day for 5 days which resulted in an increase in the concentration of intracellular ROS and decreased mitochondrial membrane potential (data not shown). Pigmentation levels can affect the absorption of UV light; however, pigmentation levels between low‐ and high‐risk donors did not differ significantly (*p* = .5; Supporting Information Fig. S3D). *IL6* expression, a cytokine previously associated with AMD 56, was increased in response to UV in both low‐ and high‐risk iPSC‐RPE cells (Fig. 7A). *SOD2, VEGF, IL18, CFH*, and *FHL‐1* expression increased only in the high‐risk RPE cells (Fig. 7B–7E), suggesting inflamasome activation. The expression of *CFI* was upregulated only in the low‐risk iPSC‐RPE cells (Fig. 7F). No change in *APOE, TNF‐α, IL1β, CFH, C3, C5, RPE65*, or *OTX2* expression was observed in both high and low‐risk iPSC‐RPE upon UV treatment (Fig. 7G–7N). Mitochondrial area decreased in both low‐ and high‐risk iPSC‐RPE when exposed to 390–410 nm UV light (Fig. 7P), while the overall number of mitochondria remained similar (Fig. 7Q). While there was no significant increase in the number of vacuoles or drüsen‐like deposits in low‐risk iPSC‐RPE cells, in the high‐risk iPSC‐RPE both parameters decreased significantly (below levels observed in the low‐risk iPSC‐RPE cultures; Fig. 7R, 7S; Supporting Information Fig. S3E), which we hypothesize is possibly due to increased expression of *SOD2* and other protective complement proteins (FHL‐1, CFH) in response to UV exposure. Photobiomodulation or optogenetics has shown that specific wavelengths of light exhibit physiological effects on biological systems. Near‐infrared, for example, enhances mitochondrial activity via activation of cytochrome oxidase [a photoacceptor, due to four redox active metal centres, presumed to convert bosons (photons) to fermions (electrons and positrons)], increases in electrons leads accelerated electron transport and increased generation of ATP 57. Currently, a photoacceptor, such as cytochrome oxidase, for 390–410 nm light has yet to be identified. Traditionally, studies have focused on the “optical window” biased on absorption and scattering of light in tissues, which is higher for both in the blue end of the spectrum; however, the optical properties of the cornea decrease this scattering effect making blue light physiologically relevant to the retina. Additionally, microvilli length in high‐risk donor cells increased in response to UV (Fig. 7O). Together, our data suggest that low‐ and high‐risk iPSC‐RPE cells respond differently to UV light exposure and set precedence for using iPSC‐RPE disease modeling as a platform for testing existing and new therapeutic regimes.

## Discussion

To date, there are no effective treatments that target the underlying disease process in AMD. Availability of patient specific models which can generate large numbers of RPE cells would provide a significant advance for a better understanding of AMD physiopathology, the contribution of environmental, lifestyle, and dietary factors and drug testing. The advent of iPSC technology has made the in vitro modeling of many inherited diseases possible; however, to date, this method has predominantly been viewed as useful to physiopathologies that manifest early during development or in childhood. Three recent studies have implemented premature aging approaches to model Parkinson's disease 58 and AMD 23 using iPSC, paving the way for modeling of complex age related disease.

In this article, we investigated whether iPSC could be used to provide a disease modeling tool which mimics an AMD phenotype in the laboratory in the absence and presence of stress stimuli. Our rationale was to focus on patients phenotyped with a significant risk factor for AMD, such as Y402H polymorphism in the *CFH* gene. Using the iPSC derived RPE cells generated from low‐ and high‐risk donors in the absence of any stress stimuli, we have been able to confirm several key cellular features of AMD as follows: (a) increased expression of inflammatory markers (for example *IL1β*); (b) lower expression of the protective oxidative stress markers (*SOD2*); (c) increased number of stress vacuoles (and their surface area); (d) increased accumulation of lipid droplets; and (e) increased expression of LC3 vesicles and higher p62 expression/aggregate suggestive of impaired autophagy. Most importantly, we were able to identify the formation of deposits comprising of components including Apolipoprotein E and C5b‐9, in keeping with drüsen formation. These deposits occupied a significantly higher volume in the RPE derived from high‐risk lines. The presence of drüsen and its larger volume in RPE derived from the high‐risk iPSC lines, together with confirmation of key molecular features observed in previous AMD studies, suggest that this iPSC model closely mimics the disease phenotype observed in AMD patients.

The complement proteins associated with AMD (FH, FI, FHR1, FHR3, C2, and C3) are plasma proteins predominantly produced in the liver; however, biosynthesis at extrahepatic sites is now well recognized 17, 18. As with the blood–brain barrier, the blood–retinal barrier limits access to circulating plasma proteins, and it has been suggested that local complement synthesis may be required for its effects in such areas. Indeed, it has been shown that choroid and RPE as well as cultured unstimulated RPE cells produce transcripts for most classical and alternative pathway complement genes 17, 18. Data summarized in this manuscript indicate that iPSC derived RPE cells express the active complement proteins and are able to modulate their expression in response to stress stimuli without having to rely on secretion from the choroid and diffusion through the BrM as suggested previously 18.

In view of this local complement regulation by RPE cells themselves, we were interested to assess the response of low‐ and high‐risk RPE responses to stress stimuli. Since a trend toward an association between severity of light exposure and AMD has been suggested by epidemiological studies, we exposed the iPSC‐derived RPE to repeated doses of UV for 5 consecutive days. Pigmentation level between the two groups was not significantly different. The low‐risk RPE cells responded by increasing the expression of inflammatory marker *IL6* and *CFI*. More significant changes were observed in high‐risk RPE cells, which upregulated the expression of protective oxidative stress defense protein, *SOD2*, as well as *CFH* and its truncated form, *FHL‐1*, in addition to showing an improved ultrastructural (increased microvilli length, reduced number of stress vacuoles, and lower mitochondrial area) and functional (lower volume of drüsen‐like deposits) properties. These results indicate that the low‐ and high‐risk AMD‐RPE cells respond very differently to UV exposure and moreover this provides evidence for UV mediated functional and cellular improvement of AMD‐associated cellular changes in high‐risk AMD‐RPE cells. These intriguing results which we attribute to increased *SOD2* expression need to be validated in a larger number of cell lines derived from additional high‐risk donors over longer intervals and with different UV doses. They do, however, highlight an important role for increased oxidative stress defense as a potential therapeutic strategy for AMD, corroborating recent data obtained with the HTRA1/ARMS2‐iPSC model and exposure to nicotinamide 25.

Several clinical trials attempting to inhibit the complement pathway have been completed or are under way including FCFD4514S (anti‐CFD [complement factor D]), LFG 316 (anti‐C5), ARC1905 (anti‐C5), catalyst protease (anti‐C3), and eculizimab (anti‐C5) 59, 60. In particular, Lampalizumab (FCFD4514S) has been shown to reduce the geographic atrophy enlargement in phase II trials of dry AMD in patients who also have a *CFI* polymorphism, indicating that inhibition of complement is a promising approach. Nonetheless, human clinical trials are complex, expensive, and require prolonged periods to assess the long‐term effect of a therapy in large numbers of patients with specific phenotypes to provide a consistent end point. The assessment is further complicated by differing progression rates in patients and the uncertain choice of disease endpoints to assess progression 61. This is a significant problem and can lead to trials having negative but disputed conclusions (e.g., the COMPLETE study on Eculizimab for dry AMD) 62. A robust and well characterized in vitro model such as the one described herein provides an efficient tool to assess potential therapeutic agents to treat AMD (such as complement pathway modulation), to better understand disease physiopathology and to test/repurpose drugs.

## Author Contributions

D.H.: experimental design, analysed data, performed research and prepared manuscript, final approval of manuscript; J.C.: fund raising, experimental design and performed research, final approval of manuscript; S.B., V.C., A.B., L.L., E.G.O., and G.A.: performed research, final approval of manuscript; Y.X.: analysed data, final approval of manuscript; C.M., A.L., M.M.K., D.S., and D.K.: fund raising and experimental design, final approval of manuscript; S.P.: performed research, analysed data and contributed to manuscript writing, final approval of manuscript; S.A.: performed research and analysed data, final approval of manuscript; V.K.: experimental design, performed research and data analysis, final approval of manuscript; G.S.: experimental design and performed research, final approval of manuscript; L.A.: designed research and fund raising, contributed to manuscript writing final approval of manuscript; M.L.: designed and performed research, prepared manuscript and fund raising, final approval of manuscript.

## Disclosure of Potential Conflicts of Interest

The authors indicated no potential conflicts of interest.

## Supporting information

Supplementary Figure 1

Supplementary Figure 2

Supplementary Figure 3

Supplementary Figure 4

Supplementary Figure 5
