# Supplementary figures and images for: An Induced Pluripotent Stem Cell Patient Specific Model of Complement Factor H (Y402H) Polymorphism Displays Characteristic Features of Age‐Related Macular Degeneration and Indicates a Beneficial Role for UV Light Exposure
Source: Stem Cells. 2017 Oct 9;35(11):2305–20. doi: 10.1002/stem.2708 (PMC5698780; doi:10.1002/stem.2708)

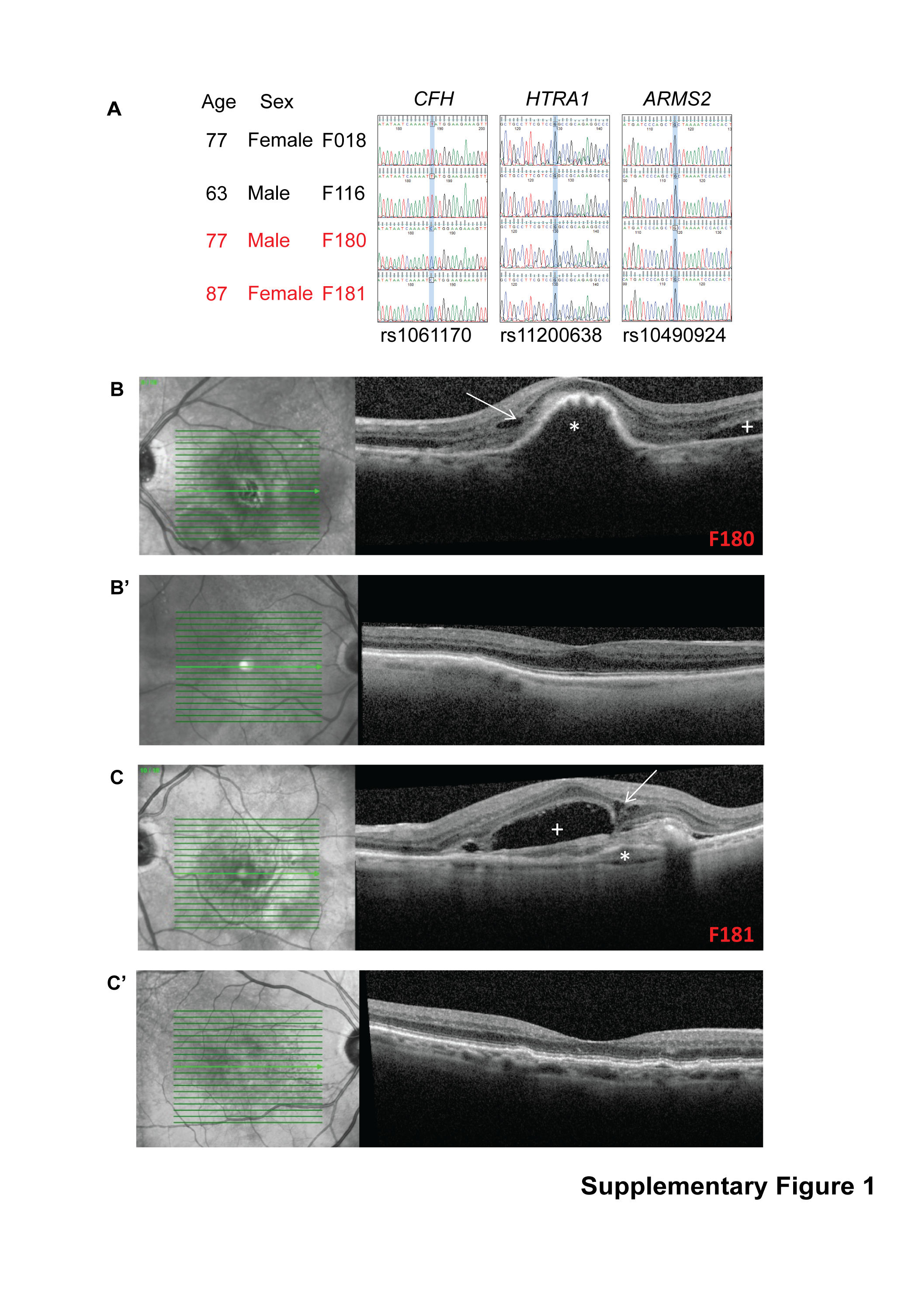

Supplement: Supplementary file 1 — Supplementary Figure 1 [file STEM-35-2305-s001.tif]

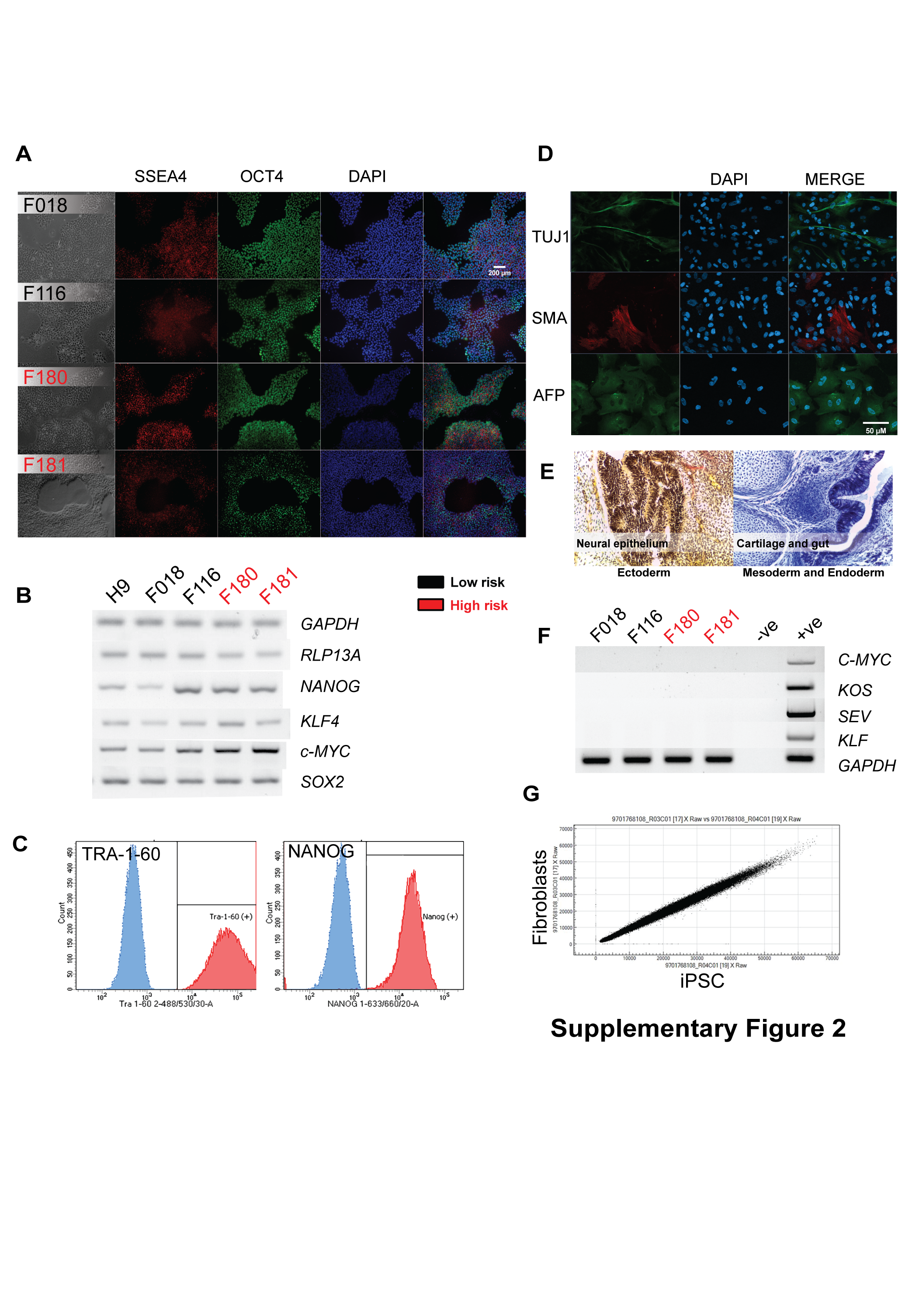

Supplement: Supplementary file 2 — Supplementary Figure 2 [file STEM-35-2305-s002.tif]

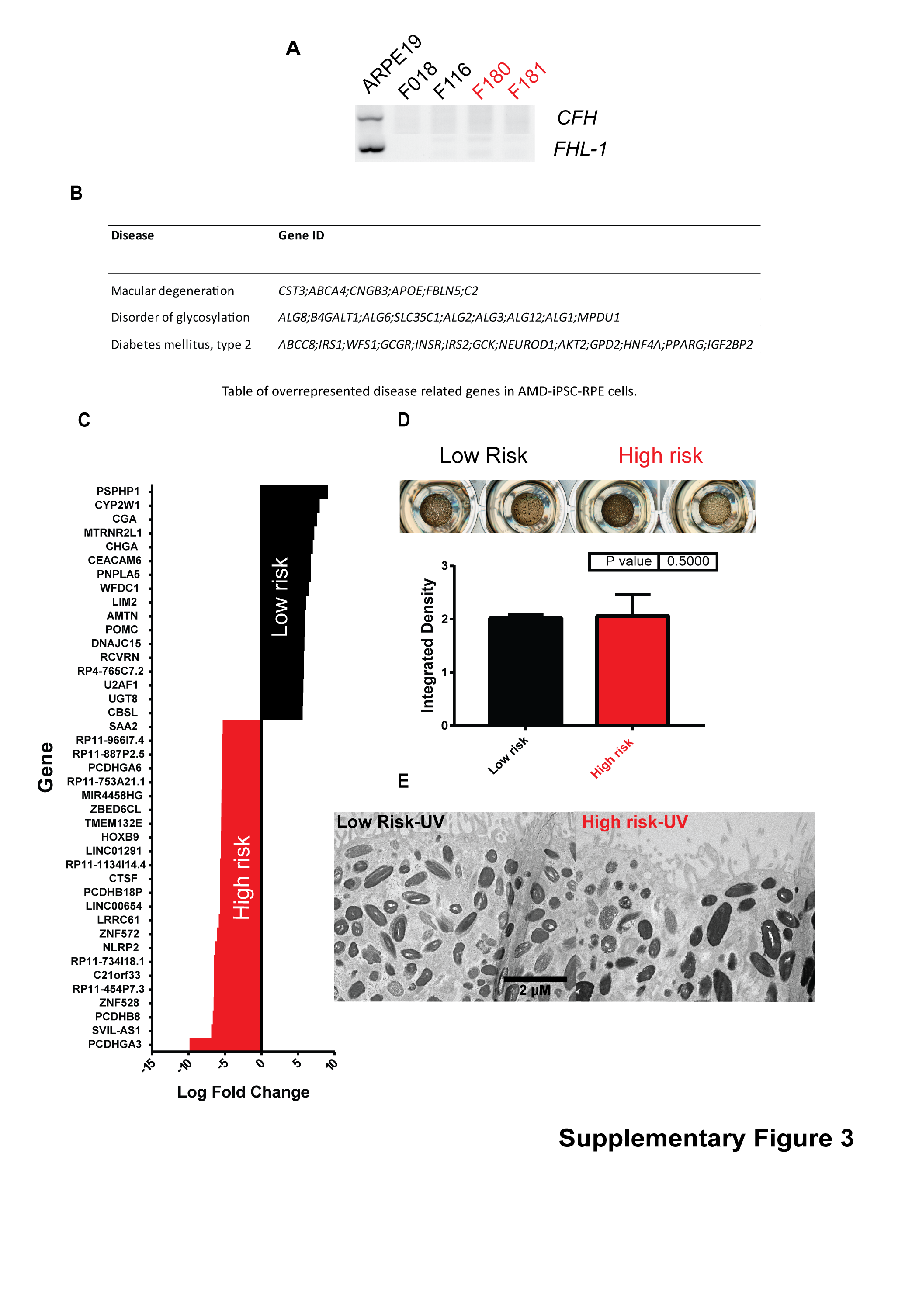

Supplement: Supplementary file 3 — Supplementary Figure 3 [file STEM-35-2305-s003.tif]

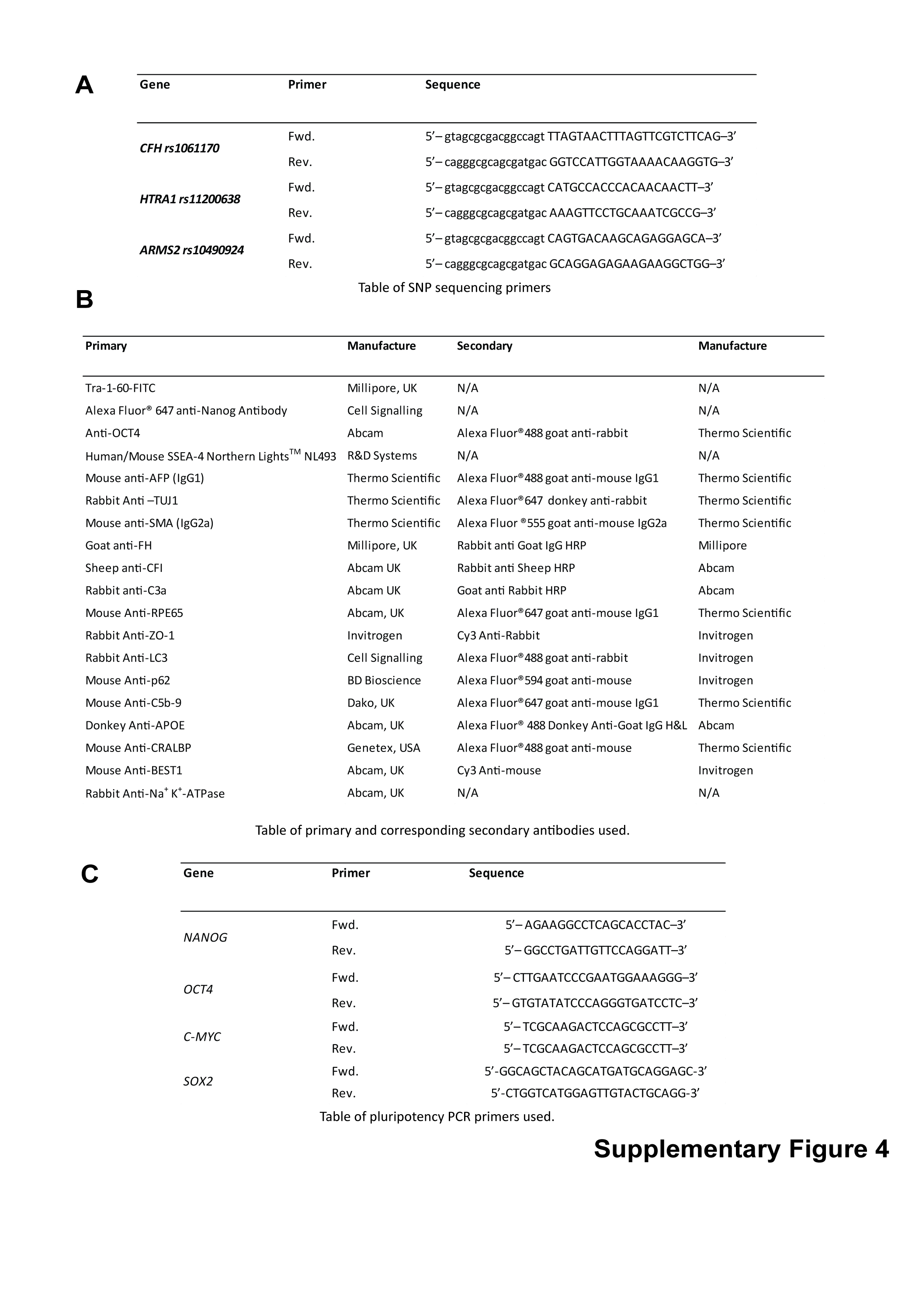

Supplement: Supplementary file 4 — Supplementary Figure 4 [file STEM-35-2305-s004.tif]

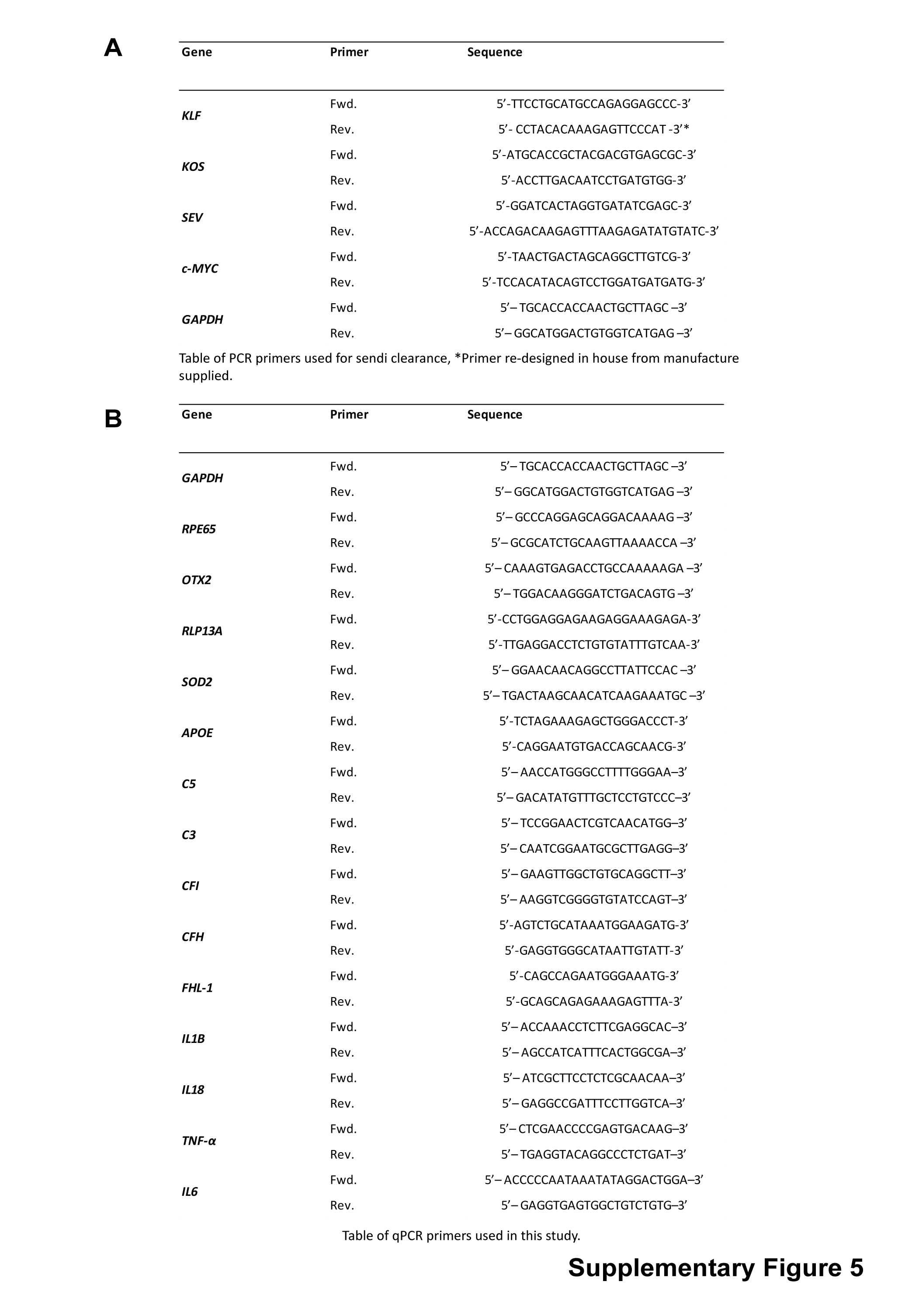

Supplement: Supplementary file 5 — Supplementary Figure 5 [file STEM-35-2305-s005.tif]
